# A Comparison of Mental Health among Earthquake, Tsunami, and Nuclear Power Plant Accident Survivors in the Long Term after the Great East Japan Earthquake

**DOI:** 10.3390/ijerph192114072

**Published:** 2022-10-28

**Authors:** Noriko Sodeyama, Sho Takahashi, Miyuki Aiba, Yayoi Haraguchi, Tetsuaki Arai, Hirokazu Tachikawa

**Affiliations:** 1 Department of Psychiatry, Faculty of Medicine, University of Tsukuba, 1-1-1 Tennodai, Tsukuba 305-8577, Japan; 2 Department of Disaster and Community Psychiatry, Faculty of Medicine, University of Tsukuba, 1-1-1 Tennodai, Tsukuba 305-8577, Japan; 3 Faculty of Human Sciences, Toyo Gakuen University, 1-26-3 Hongo, Bunkyo-ku, Tokyo 113-0033, Japan; 4 College of Humanities and Social Sciences, Ibaraki University, 2-1-1 Bunkyo, Mito 310-8512, Japan

**Keywords:** depression, post-traumatic stress, risk factor, regional difference, the Great East Japan Earthquake

## Abstract

Even now, ten years after the Great East Japan Earthquake (GEJE), thousands of residents of Fukushima Prefecture are still living as evacuees. To understand the mental health states and needs for psychological care of the survivors, we conducted a questionnaire survey of survivors who were evacuated from Fukushima to Ibaraki due to the nuclear power plant accident and of the residents of two areas in Ibaraki where damage was particularly severe due to the tsunami or liquefaction. Our results show that stress related to participants’ hometowns and to the disaster was a risk factor for depression and post-traumatic stress among the survivors in the medium to long term in all regions examined. Other risk factors for post-traumatic stress differed by region. This study shows that in a complex disaster such as the GEJE, where damage is widespread, the causes of damage and the experiences of disaster survivors differ greatly from region to region and that risk factors for depression and post-traumatic stress among disaster survivors over the medium to long term may also differ. To provide appropriate care to disaster survivors, it is necessary to determine what is causing the risk of depression and post-traumatic stress at any given time and in specific regions.

## 1. Introduction

The Great East Japan Earthquake (GEJE), which occurred in 2011, was a complex disaster caused by a massive magnitude 9.1 earthquake, the resulting tsunami, liquefaction, and a nuclear power plant accident. The damage resulting from the GEJE was very widespread. As a result, the damage situation varied greatly from region to region. A total of 400,000 houses were destroyed [1]; about 16,000 people died, of which about 14,000 drowned in the tsunami [2]; the number of evacuees just after the disaster was over 340,000 [3], of which more than 170,000 were long-term evacuees from Fukushima Prefecture under the evacuation order issued due to the Fukushima Daiichi nuclear accident [4], and of these, 70,000 were evacuated to other prefectures in Japan [5]. As of 2016, five years after the GEJE, the evacuation order around the Fukushima Daiichi Nuclear Power Plant remained in place, and 40,000 people continued as evacuees outside Fukushima [5].

Regional differences in mental status among disaster survivors have been reported. Surveys two years after the 1988 Armenia earthquake [6] and one to two months after the 2017 Mexico earthquake [7] found that the highest prevalence of post-traumatic stress disorder (PTSD) was observed in the state with more material damage caused by the earthquake. A study 18 months after the 1999 Izmit earthquake reported that rates of PTSD and depression were higher in the site closer to the epicenter [8]. A survey seven to eight months after the 2008 Sichuan earthquake also showed that the predictive factor of PTSD symptoms included proximity to the epicenter [9]. However, there are few reports examining regional differences in mental health in complex and widespread disasters, including evacuees from the affected areas.

In the aftermath of the Fukushima Daiichi nuclear accident that followed the GEJE, approximately 4000 Fukushima residents were evacuated to the neighboring Ibaraki Prefecture, which is located about 100 km south of the nuclear power plant. In Ibaraki, liquefaction occurred in southern coastal cities in addition to the earthquake and tsunami, which occurred in northern coastal cities due to the GEJE. There were 778 casualties (including missing persons), and approximately 28,000 houses were destroyed [10]. Regional differences in the mental conditions of the survivors of the GEJE have not yet been fully examined. Grimm et al. reported that the type of disaster is a significant predictor of post- and peri-traumatic outcomes [11]. Reifels et al. suggest that the type of disaster should be considered when tailoring psychosocial responses to a disaster [12]. We hypothesize that in a complex disaster with widespread damage such as the GEJE, there are differences in the causes of damage and the experiences of disaster survivors by region, and differences in risk factors for depression and post-traumatic stress in survivors. It is important to understand the medium-(approximately 1 to 3 months) to long-term (approximately 4 or months years) characteristics of the mental health of survivors, as they pertain to a disaster situation, from the viewpoint of caring for the survivors.

To understand regional differences in the mental health states and needs for psychological care of survivors, in the medium to long term after the GEJE, a widespread disaster, we conducted a questionnaire survey of survivors who were evacuated from Fukushima to Ibaraki due to the nuclear power plant accident and of the residents of two areas in Ibaraki where damage was particularly severe due to the tsunami or liquefaction. We previously reported that long-term evacuees living outside of Fukushima Prefecture were still suffering from mental disorders five years after the disaster [13]. In the present study, we further compared the mental health characteristics in survivors located in three different regions that each experienced different disaster situations.

## 2. Materials and Methods

### 2.1. Participants

For this study, surveys were sent to 1470 households that were established in Fukushima when the GEJE occurred and evacuated to Ibaraki by October 2016. In addition, municipalities randomly selected 1500 households present, as of October 2016, in Kitaibaraki City and Kamisu City, Ibaraki and sent them surveys for this study.

**Figure 1:**
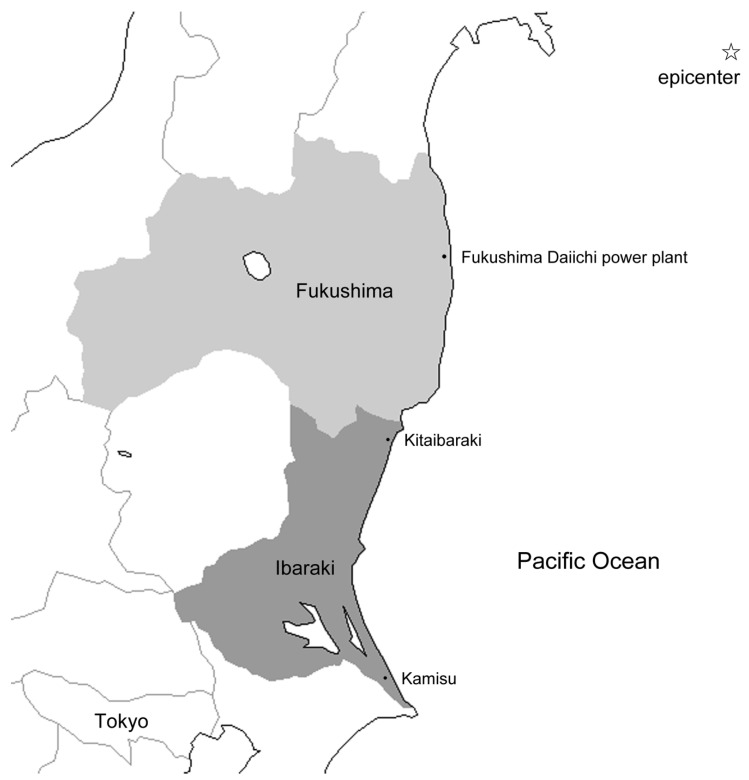
Area map.

Of the three locations, Fukushima is the closest to the epicenter (38°06′ N 142°51′ E) [14] and Kamisu is the farthest (Figure 1). Kitaibaraki is located approximately 75 km south of the Fukushima Daiichi nuclear power plant (37°42′ N 141°03′ E), on the northern coast of the prefecture, and was seriously damaged by the tsunami caused by the GEJE. In Fukushima, the number of casualties was 1810 (including missing persons), and approximately 98,218 houses were destroyed (Table 1). The inundation height resulting from the tsunami at the coastal city of Kitaibaraki was estimated to be 6.9 m, and fishing ports suffered enormous damage to industrial infrastructure, such as the destruction and outflow of fishing boats and fishery processing facilities. The number of casualties was 199 (including missing persons), and approximately 1513 houses were destroyed. Kamisu, which is about 175 km south of the Fukushima Daiichi nuclear power plant, is located on the southern end of the Ibaraki coast. Kamisu suffered great damage to its houses due to liquefaction in addition to the flood damage caused by the tsunami. The number of casualties was 6 (including missing persons), and approximately 1949 houses were destroyed [10].

### 2.2. Survey Methods

A voluntary questionnaire was sent to all households who were evacuated from Fukushima to Ibaraki. A non-profit organization (NPO) called “*Fūai-net*,” which is designated as a support center for livelihood reconstruction, assisted in the distribution of the questionnaire by including copies in their regular mailings sent to all Ibaraki households via local governments. Questionnaires were also mailed directly to households in both Kitaibaraki and Kamisu, Ibaraki. Residents (one adult per household) who chose to participate returned completed questionnaires by mail. The survey was conducted between October 2016 and January 2017.

As described previously [13], the survey questions included the following areas:

(1) Basic attributes of the participants:
  Age (20–59 and over 60 years of age), gender (male, female), and educational background (elementary/junior high school, senior high school, vocational/junior college, university/graduate school, and other)
(2) Degree of damage:
  Causes of damage (the earthquake, the tsunami, the nuclear accident, and harmful rumors) and details regarding damage (own injuries/illness, death of/missing family members or friends, complete/partial destruction of house, loss of employment, and family separation/discord)
(3) Current health condition: causes of stress during the past month
  (a) Economic problems: bankruptcy, bad business performance, debt, poverty, and unemployment
  (b) Work-related problems: job changes, poor work performance, and relationship problems at workplaces
  (c) Neighborhood problems: discord or isolation
  (d) Worries about hometown (evacuees from Fukushima only): condition of their house in Fukushima, the possibility of returning home, and anxiety about nuclear-related issues
  (e) Worries about the disaster (Kitaibaraki and Kamisu only): changes in livelihood due to the GEJE, problems with nuclear power plants, and anxiety about future disasters

Further, the mental conditions of the survey takers were examined from two perspectives (depression as severe psychological distress and post-traumatic stress) using psychological assessment scales. Participants also had the opportunity to include their own comments at the bottom of the questionnaire. Finally, information about mental health resources, including contact information, was provided and participants were urged to seek support if they needed it.

### 2.3. Psychological Assessment

The mental conditions of survey takers were assessed using the Kessler Psychological Distress Scale (K6). The K6, which assesses the degree of psychological distress, was developed by Kessler et al. [15]. It is a 6-item questionnaire that uses a 5-point rating scale to evaluate psychological distress experienced during the past month. The Japanese version developed by Furukawa et al. [16] was used. Higher scores (13 and above; overall possible range: 0–24) indicate a greater chance of mood or anxiety disorders [15], including depression.

The Impact of Events Scale-Revised (IES-R), which was adapted by Weiss et al. [17] based on the original version by Horowitz et al. [18], was used to examine Post-traumatic stress symptoms (PTSS). The Japanese version, developed by Asukai et al. [19] was used. Using 22 items, the IES-R gauges three relevant symptoms: Intrusion, Avoidance, and Hyperarousal. The items were developed based on the Diagnostic and Statistical Manual of Mental Disorders, Fourth Edition (DSM-IV). Survey takers used a 5-point scale to answer questions based on their condition during the past seven days. Scores range from 0–88, and higher scores (25 and above) suggest a higher possibility of post-traumatic stress [20].

### 2.4. Statistical Analysis

SPSS Ver.26.0 was used for statistical analysis. First, participants were divided into two groups: 1. high risk for depression (cut-off value of 13 points or more on the K6) and 2. high risk for PTS (cut-off value of 25 points or more on the IES-R). Second, question-item ratios were compared using a chi-square test. Third, binomial logistic regression analysis (forced entry method) was conducted for items that reached significance in the chi-square test (*p* < 0.05) as independent variables and the presence of a high risk of depression or a high risk of post-traumatic stress as dependent variables. Finally, we calculated the odds ratio (OR), which is an index of relative risk, and the 95% confidence interval.

### 2.5. Ethical Considerations

The surveys were conducted anonymously in order to protect personal information. The purpose and methods of the study were explained to the participants. Informed consent was assumed when the completed questionnaire was returned. Approval of this study was obtained from the medical ethics committee of the University of Tsukuba (No. 1094).

## 3. Results

### 3.1. Survivors’ Attributes, Damage Conditions, and Psychological Symptoms

Responses were received from 310 (156 male; 151 female) out of 1470 potential participants (21.1%) among evacuees from Fukushima, 371 (165 male; 205 female) out of 1500 potential participants (24.7%) in Kitaibaraki, and 402 (176 male; 223 female) out of 1500 potential participants (26.8%) in Kamisu. The total response rate was 24.2%. Regarding age, 52.9% of the evacuee respondents were aged 60 and over, 47.3% in Kitaibaraki, and 37.0% in Kamisu. The chi-square test for the proportion by gender in the three regions showed no significant difference (*p* = 0.156), while the standardized residuals for the proportion of those aged 60 and over in the three regions were 3.3 for those who evacuated from Fukushima and −4.1 for Kamisu, indicating a significant difference (*p* < 0.01).

The characteristics of the survivors with psychological distress are shown in Table 2 and those of survivors with post-traumatic stress symptoms are shown in Table 3.

As for the cause of the damage, the largest number of evacuees from Fukushima answered that it was the ‘nuclear accident’ (287; 92.6%), followed by the ‘earthquake’ (243; 78.4%). In Kitaibaraki, the largest number of respondents answered ‘earthquake’ (320; 86.3%), followed by ‘tsunami’ (109; 29.4%). In Kamisu, the most common answer was ‘earthquake’ (297; 73.9%), followed by ‘liquefaction’ (89; 22.1%).

#### 3.1.1. High Risk for Depression as Severe Psychological Distress (K6 ≥ 13)

The number of participants with a high risk for depression as severe psychological distress (K6 ≥ 13) was 51 (16.5%) among evacuees from Fukushima, 33 (8.9%) in Kitaibaraki, and 25 (6.2%) in Kamisu.

The percentage of participants with a high risk of depression was significantly higher among the following groups and conditions:

Evacuees from Fukushima
  Causes of damage
    Not significant (N.S.)
  Damage
    Being injured or ill because of the disaster
  Causes of stress in the past month
    Work-related problems
    Neighborhood problems
    Worries about their hometown/disaster
Kitaibaraki
  Causes of damage
    Tsunami
  Damage
    Being injured or ill because of the disaster
    Deaths of or missing family members or friends
    Having their houses partially destroyed
    Losing their jobs
  Causes of stress in the past month
    Economic problems
    Neighborhood problems
    Worries about their hometown/disaster
Kamisu
  Age (≥ 60)
  Causes of damage
    N.S.
  Damage
    N.S.
  Causes of stress in the past month
    Economic problems
    Work-related problems
    Worries about their hometown/disaster

#### 3.1.2. High Risk for Post-Traumatic Stress (IES-R ≥ 25)

The number of participants with a high risk for post-traumatic stress (IES-R ≥ 25) was 121 (39.0%) among evacuees from Fukushima, 87 (23.4%) in Kitaibaraki, and 84 (20.8%) in Kamisu.

The percentage of participants with a high risk of post-traumatic stress was significantly higher among the following groups and conditions:

Evacuees from Fukushima
  Causes of damage
    Harmful rumors
  Damage
    Being injured or ill because of the disaster
    Deaths of or missing family members or friends
    Separation or discord among family members
  Causes of stress in the past month
    Economic problems
    Work-related problems
    Neighborhood problems
    Worries about their hometown/disaster
Kitaibaraki
  Causes of damage
    Tsunami; Harmful rumors
  Damage
    Being injured or ill because of the disaster
    Having their house partially destroyed
    Separation or discord among family members
  Causes of stress in the past month
    Economic problems
    Neighborhood problems
    Worries about their hometown/disaster
Kamisu
  Causes of damage
    Earthquake; Liquefaction
  Damage
    Deaths of or missing family members or friends
    Having their house partially destroyed
    Losing their jobs
  Causes of stress in the past month
    Economic problems
    Work-related problems
    Worries about their hometown/disaster

### 3.2. Factors Affecting Psychological Conditions of Survivors

Correlations between depression as severe psychological distress, as well as post-traumatic stress, and age, gender, and significant items revealed by the chi-square test were examined using binomial logistic regression analysis. Among the significant items revealed by the chi-square test, those that included cells with an expected value of less than 5 were excluded because they were small, and the analysis would be unstable (Table 4 and Table 5).

First, we examined correlations with depression among the three regional groups. Among the evacuees from Fukushima, ‘getting injured or ill because of the disaster’ (Odds Ratio [OR]: 5.73, 95% Confidence Interval [95% CI]: 2.53–12.99), ‘having work-related problems’ (OR: 4.23, 95% CI: 1.69–10.57), and ‘worries about their hometown/disaster’ (OR: 4.36, 95% CI: 1.95–19.42) were significant risk factors for depression. In Kitaibaraki, ‘having experienced the deaths of or missing family members or friends’ (OR: 4.93, 95% CI: 1.25–19.42) and ‘worries about their hometown/disaster’ (OR: 4.43, 95% CI: 1.80–10.90) were significant risk factors. In Kamisu, ‘having economic problems’ (OR: 3.82, 95%; CI: 1.53–9.57) and ‘worries about their hometown/disaster’ (OR: 4.80, 95% CI: 1.34–17.21) were significant risk factors.

Next, we examined the correlations with post-traumatic stress symptoms among the three regional groups. Among the evacuees from Fukushima, damage caused by ‘harmful rumors’ (OR: 2.60, 95% CI: 1.22–5.55), ‘getting injured or ill because of the disaster’ (OR: 3.61, 95% CI: 1.52–8.57), ‘neighborhood problems’ (OR: 3.69, 95% CI: 1.67–8.17), and ‘worries about their hometown/disaster’ (OR: 4.13, 95% CI: 2.29–7.47) were significant risk factors for post-traumatic stress. In Kitaibaraki, ‘having their houses completely destroyed’ (OR: 3.49, 95% CI: 1.81–6.72) and ‘worries about their hometown/disaster’ (OR: 3.49, 95% CI: 1.81–6.72) were significant risk factors. In Kamisu, having ‘economic problems’ (OR: 2.46, 95% CI: 1.33–4.56) and ‘worries about their hometown/disaster’ (OR: 4.26, 95% CI: 1.61–11.24) were significant risk factors.

## 4. Discussion

### 4.1. Psychological Symptoms among Survivors of the GEJE

The results of this survey identified conditions and risk factors for depression as severe psychological distress and post-traumatic stress among survivors five years after the complex GEJE, tsunami, and nuclear accident. The findings are uniquely important because there are no reports comparing the medium- to long-term mental health of evacuees from Fukushima and survivors in other prefectures after the GEJE. The strength of this study is that, in a complex disaster with widespread damage such as the GEJE, it shows differences in the causes of damage and the experiences of disaster survivors by region. It also explores differences over the medium-(approximately 1 to 3 months) to long-term (approximately 4 or more months) risk factors for depression and post-traumatic stress in survivors, as discussed below, by comparing results from earlier studies to those from the present study.

It has been reported that the percentage of Japanese people with a cut-off value of over 13 points on the K6 is 3% during normal times [21]. The percentage of participants with a K6 score of 13 or higher was highest among evacuees from Fukushima, at 16.5%, which is 5.5 times higher than normal and 2.7 times higher than that of participants in Kamisu. This suggests that more evacuees from Fukushima were suffering from depression in the medium to long term after the GEJE than were participants in other areas.

The survey conducted by Sato et al. [22] two years after the disaster of evacuees from Fukushima to Ibaraki reported that the percentage of participants with a cut-off value of over 25 on the IES-R was 53.2%. The current survey, conducted five years after the disaster, indicates that this percentage had decreased to 39.0%. In a survey conducted in Kitaibaraki two years after the GEJE, the percentage of people whose IES-R was above the cut-off value was 32.7% [22], but in the current survey, conducted five years after the disaster, it was 23.4%, also decreasing. However, even in Kamisu, where the proportion of people with IES-R values above the cut-off is the lowest among the three surveyed areas, 20.8% of people may still have post-traumatic stress symptoms. It is considered a serious problem that many people were continuing to suffer from post-traumatic stress symptoms five years after the disaster.

In this study, the proportion of people with symptoms of depression or post-traumatic stress was higher the closer their residential area was to the epicenter at the time of the disaster. The results for post-traumatic stress symptoms were similar to that in previous studies [6,7,8,9]. Material and human damages were also generally greater closer to the epicenter, which may have contributed to these results [6,7,9]. It is known that disaster victims have a stronger fear of human-made disasters, especially radiation disasters, compared to natural disasters [23,24]. The fact that the place of residence at the time of the disaster was closer to the Fukushima Daiichi Nuclear Power Plant as well as to the epicenter of the earthquake may have affected the mental state of the survivors. The people of Fukushima who were evacuated to different prefectures for a long period of time after the nuclear accident experienced a major change in their environments due to the evacuation, which might also impact their mental state. 

### 4.2. Risk Factors for Psychological Symptoms among Types of Survivors of the GEJE

#### 4.2.1. Risk of Depression

Tang et al. [25] identified the following risk factors for depression caused by disasters: being female, not being married, holding religious beliefs, having a poor education, having experienced prior trauma, experiencing fear, and experiencing injury and/or bereavement during the disaster. The current study examined gender, educational background, injuries, and bereavement resulting from the GEJE. ‘Getting injured or ill’ among evacuees from Fukushima and ‘having experienced the deaths of or missing family members or friends’ because of the disaster among participants in Kitaibaraki were risk factors for depression, but gender and education were not.

In our study, human damages due to disaster had the highest odds ratio among the risk factors for depression even five years after the earthquake among evacuees from Fukushima and participants in Kitaibaraki. This suggests that the experience of human damages may be a risk factor for depression even in the long term after a disaster. In Kamisu, direct damage by the disaster was not a risk factor for depression. In Kamisu, which suffered the least amount of damage of the three areas, the direct effect of the disaster on depression may have almost disappeared five years after the disaster. However, having worries related to one’s hometown or the disaster was a risk factor for depression in all three affected areas. In the long term, the disaster remains a stressor for many people, especially for those evacuated from Fukushima, and this may increase the risk of depression.

#### 4.2.2. Risk of Post-Traumatic Stress

Previous studies have identified the following factors for the onset of PTSD risk: the degree of exposure to the disaster, not receiving social support after the disaster (or the feeling of lacking social support), being female, and secondary stress factors [26]. In our results, too, secondary stressors such as exposure to the disaster through one’s own injury or illness, complete destruction of one’s house, economic problems, work-related problems, and neighborhood problems were risk factors for post-traumatic stress.

In Kitaibaraki, the complete destruction of one’s house due to the disaster had the highest odds ratio among the risk factors for post-traumatic stress. However, among the evacuees outside Fukushima, the complete destruction of one’s house because of the disaster was not a risk factor. Since the evacuees from Fukushima had to live away from their homes due to the evacuation order, the psychological impact of material damages may have been reduced. On the other hand, the odds ratio of stress due to worry about one’s hometown was the highest among evacuees from Fukushima. Harmful rumors related to the nuclear accident was also a risk factor for post-traumatic stress among them. In the long term after the GEJE, the nuclear accident was still thought to be affecting the mental health of the evacuees from Fukushima in various ways. In Kamisu, the direct damage of the GEJE was not a risk factor for post-traumatic stress or for depression. However, having “worries related to the disaster” had the highest odds ratio as a risk factor for post-traumatic stress in Kamisu. Although “worries related to the disaster” includes anxiety about future disasters, and we cannot deny the possibility that the GEJE continues to affect the mental health of Kamisu residents.

#### 4.2.3. Provision of Long-Term Care

First, more attention should be paid to those who have been injured or become ill because of the disaster, as well as to those who have experienced the deaths of or missing family members or friends. Second, the extent of material damage is also likely to be linked to the risk of post-traumatic stress. Therefore, economic support as well as psychological care is essential for those who have experienced major material damage, such as the complete destruction of their houses. In the case of the nuclear power plant accident, which is a unique human disaster, it is important to provide support with regard to harmful rumors. Financial compensation is needed for the economic losses caused by harmful rumors, and other support such as social awareness is needed to counteract or prevent psychological damage. The percentage of evacuees from Fukushima with symptoms of depression was more than twice that of other areas, so focused care for them will be necessary in the future.

Outside of Fukushima Prefecture, there are currently no fixed-point, mid- to long-term surveys of the mental health of disaster survivors. We believe that administrative action taken directly by the municipality, rather than leaving such action up to the central government, is necessary in order to conduct surveys, such as those used in this study, in each municipality and to provide appropriate support on a municipal basis.

### 4.3. Limitations

There are several limitations in this study. First, the study was a cross-sectional study conducted in 2016. Therefore, the results of the study, including the mental state of the participants, may be different in 2022. However, the results of this study may help first responders, mental health professionals, and others who interact with survivors of future disasters to develop action plans to provide the physical, emotional, and psychological support that survivors need. Second, because the study was cross-sectional, the causality between the core outcomes and the independent variables used in the analysis remains unclear. We need the approval of the municipality and the non-profit organization before we can conduct a second measurement equivalent to the first. We believe that it would be valuable to conduct a second measurement with the cooperation of the municipality and analyze the scores of the psychological assessment scales as quantitative data to see the evolution of depression and specific post-traumatic symptoms. Third, the response rate of the study was rather low, at 24.2%. Therefore, the proportion of people with symptoms in this study should be carefully evaluated. The response rate may have been low because people wanted to avoid recalling the painful events of the disaster. Fourth, respondents were limited to one person that was randomly selected from each household. Therefore, people having severe psychological symptoms or feelings of resistance to getting support might have been excluded from responding. Furthermore, it is possible that only the people who were interested in the psychological and social problems related to the GEJE might have participated in the survey. It is also possible that participants already had some knowledge of or experience with mental illness. This may have decreased the ability of this study to allow certain assumptions about the reasons for perceived psychological and social problems. Fifth, this study examined regional differences in psychological symptoms among disaster survivors in a widespread disaster. However, since only participants from Fukushima were long-term evacuees, the differences between their psychological symptoms and those of the residents of Kitaibaraki and Kamisu do not necessarily reflect the differences in the disaster situation among the regions. Finally, the age ratio of the participants varied by region, which may have affected the results.

## 5. Conclusions

We compared the psychological state of survivors in three regions with different disaster conditions five years after the GEJE. Our results show that stress related to their hometown and the disaster was a risk factor for depression and post-traumatic stress of the survivors in the medium to long term in all regions. In addition, while human damage was a risk factor for depression, the risk factors for post-traumatic stress differed by region. This study shows that in a complex disaster such as the GEJE, where damage is widespread, the causes of damage and the experiences of disaster survivors differ greatly from region to region, and that risk factors for depression and post-traumatic stress among disaster survivors over the medium to long term may also differ. To provide appropriate care for disaster survivors, it is necessary to determine what is causing the risk of depression and post-traumatic stress at a given time and in a given region. A longitudinal survey is necessary in the hardest hit areas.

## Figures and Tables

**Table 1:** Damage from the Great East Japan Earthquake.

	Fukushima Prefecture	Kitaibaraki City	Kamisu City
Population (March 2011)	2,024,401	46,789	94,932
Number of casualties	1810	199	6
Number of houses destroyed	98,218	1513	1949

**Table 2:** The Kessler Psychological Distress Scale (K6) of three regional groups depending on the participants’ attributes and damage conditions.

	Fukushima Prefecture			Kitaibaraki City			Kamisu City		
	K6 ≥ 13	K6 < 13	*p*-Value	K6 ≥ 13	K6 < 13	*p*-Value	K6 ≥ 13	K6 < 13	*p*-Value
	n (%)	n (%)		n (%)	n (%)		n (%)	n (%)	
Total	51 (16.5)	236 (76.1)		33 (8.9)	328 (88.2)		25 (6.2)	370 (91.8)	
Age									
20–59	25 (49.0)	116 (49.2)	0.986	20 (60.6)	173 (52.7)	0.388	22 (88.0)	228 (61.8)	<0.05
≥60	26 (51.0)	120 (50.8)		13 (39.4)	155 (47.3)		3 (12.0)	141 (38.2)	
Gender									
Male	22 (43.1)	123 (52.3)	0.233	17 (51.5)	146 (44.6)	0.45	12 (48.0)	163 (44.3)	0.718
Female	29 (56.9)	112 (47.7)		16 (48.5)	181 (55.4)		13 (52.0)	205 (55.7)	
Educational background									
Elementary/Junior high school	5 (10.0)	21 (9.0)	0.1	4 (12.1)	44 (13.5)	0.556	1 (4.0)	58 (15.7)	0.582
Senior high school	28 (56.0)	132 (56.4)		15 (45.5)	142 (43.4)		13 (52.0)	184 (49.9)	
Vocational/Junior college	13 (26.0)	45 (19.2)		10 (30.3)	77 (23.5)		7 (28.0)	76 (20.6)	
University/Graduate school	1 (2.0)	31 (13.2)		2 (6.1)	52 (15.9)		3 (12.0)	38 (10.3)	
Causes of damage									
Earthquake									
Yes	43 (84.3)	185 (78.4)	0.342	30 (90.9)	281 (85.9)	0.427	21 (84.0)	273 (73.8)	0.257
No	8 (15.7)	51 (21.6)		3 (9.1)	49 (14.1)		4 (16.0)	97 (26.2)	
Tsunami									
Yes	5 (9.8)	29 (12.3)	0.619	15 (45.5)	91 (27.8)	<0.05	6 (24.0)	58 (15.7)	0.274
No	46 (90.2)	207 (87.7)		18 (54.5)	236 (72.2)		19 (76.0)	312 (84.3)	
Nuclear accident									
Yes	48 (94.1)	219 (92.8)	0.737	9 (27.3)	70 (21.4)	0.438	1 (4.0)	17 (4.6)	0.89
No	3 (5.9)	17 (7.2)		24 (72.7)	257 (78.6)		24 (96.0)	353 (95.4)	
Liquefaction									
Yes	1 (2.0)	4 (1.7)	0.895	2 (6.1)	7 (2.1)	0.169	9 (36.0)	79 (21.4)	0.088
No	50 (98.0)	232 (98.3)		31 (93.9)	320 (97.9)		16 (64.0)	291 (78.6)	
Harmful rumors									
Yes	11 (21.6)	35 (14.8)	0.234	8 (24.2)	32 (13.6)	0.644	3 (12.0)	19 (5.1)	0.147
No	40 (78.4)	201 (85.2)		25 (75.8)	259 (79.2)		351 (94.9)	22 (88.0)	
Content of damage									
Own injuries/illness									
Yes	17 (33.3)	22 (9.3)	<0.01	7 (21.2)	10 (3.0)	<0.01	2 (8.0)	1 (0.3)	<0.01
No	34 (66.7)	214 (90.7)		26 (78.8)	318 (97.0)		23 (92.0)	352 (99.7)	
Death of/missing family members or friends									
Yes	11 (21.6)	43 (18.2)	0.579	5 (15.2)	13 (4.0)	<0.01	1 (4.0)	3 (0.8)	0.137
No	40 (78.4)	193 (81.8)		28 (84.8)	315 (96.0)		24 (96.0)	350 (99.2)	
Complete destruction of one’s house									
Yes	5 (9.8)	24 (10.2)	0.937	3 (9.1)	12 (3.7)	0.136	1 (4.0)	4 (1.1)	0.225
No	46 (90.2)	212 (89.8)		30 (90.9)	316 (96.3)		24 (96.0)	349 (98.9)	
Partial destructions of one’s house									
Yes	19 (37.3)	91 (38.6)	0.862	18 (54.5)	115 (35.1)	<0.05	8 (32.0)	70 (19.8)	0.146
No	32 (62.7)	145 (61.4)		15 (45.5)	213 (64.9)		17 (68.0)	283 (80.2)	
Losing a job									
Yes	14 (27.5)	95 (40.3)	0.088	5 (15.2)	19 (5.8)	<0.05	0 (0.0)	5 (1.4)	0.549
No	37 (72.5)	141 (59.7)		28 (84.8)	309 (94.2)		25 (100.0)	348 (98.6)	
Family separation/discord									
Yes	21 (41.2)	97 (41.1)	0.992	2 (6.1)	7 (2.1)	0.168	2 (8.0)	8 (2.3)	0.084
No	30 (58.8)	139 (58.9)		31 (93.9)	321 (97.9)		23 (97.0)	345 (97.7)	
Causes of stress in the past month									
Economic problems									
Yes	13 (25.5)	42 (17.8)	0.206	18 (54.5)	69 (22.0)	<0.01	12 (52.2)	60 (17.1)	<0.01
No	38 (74.5)	194 (82.2)		15 (45.5)	244 (78.0)		11 (47.8)	291 (82.9)	
Work-related problems									
Yes	17 (33.3)	35 (14.8)	<0.01	11 (33.3)	77 (24.6)	0.273	11 (47.8)	98 (27.9)	<0.05
No	34 (66.7)	201 (85.2)		22 (66.7)	236 (75.4)		12 (52.2)	253 (72.1)	
Neighborhood problems									
Yes	14 (27.5)	35 (14.8)	<0.05	6 (18.2)	12 (3.8)	<0.01	1 (4.3)	10 (2.8)	0.68
No	37 (72.5)	201 (85.2)		27 (81.8)	301 (96.2)		22 (95.7)	341 (97.2)	
Worries about one’s hometown/disaster									
Yes	41 (80.4)	108 (45.8)	<0.01	17 (51.5)	48 (15.3)	<0.01	5 (21.7)	18 (5.1)	<0.05
No	18 (29.5)	122 (51.7)		16 (48.5)	265 (84.7)		18 (78.3)	333 (94.9)	

**Table 3:** The Impact of Events Scale-Revised (IES-R) of three regional groups depending on the participants’ attributes and damage conditions.

	Fukushima Prefecture			Kitaibaraki City			Kamisu City		
	IES-R ≥ 25	IES-R < 25	*p*-Value	IES-R ≥ 25	IES-R < 25	*p*-Value	IES-R ≥ 25	IES-R < 25	*p*-Value
	n (%)	n (%)		n (%)	n (%)		n (%)	n (%)	
Total	121 (39.0)	146 (47.1)		87 (23.4)	275 (73.9)		84 (20.8)	312 (77.4)	
Age									
20–59	65 (53.7)	69 (47.3)	0.293	45 (51.7)	149 (54.2)	0.689	61 (72.6)	190 (61.1)	0.051
≥60	56 (46.3)	77 (52.7)		42 (48.3)	126 (45.8)		23 (27.4)	121 (38.9)	
Gender									
Male	65 (53.7)	71 (49.0)	0.44	34 (39.1)	127 (46.4)	0.235	33 (39.3)	141 (45.6)	0.299
Female	56 (46.3)	74 (51.0)		53 (60.9)	147 (53.6)		51 (60.7)	168 (54.4)	
Educational background									
Elementary/Junior high school	13 (10.8)	10 (6.9)	0.699	10 (11.5)	39 (14.2)	0.263	11 (13.1)	48 (15.4)	0.603
Senior high school	67 (55.8)	84 (57.9)		47 (54.0)	112 (40.9)		47 (56.0)	151 (48.6)	
Vocational/Junior college	25 (20.8)	30 (20.7)		19 (21.8)	68 (24.8)		17 (20.2)	66 (21.2)	
University/Graduate school	12 (10.0)	19 (13.1)		9 (10.3)	43 (15.7)		8 (9.5)	33 (10.6)	
Causes of damage									
Earthquake									
Yes	101 (83.5)	113 (77.4)	0.215	77 (88.5)	235 (85.8)	0.516	71 (84.5)	224 (71.8)	<0.05
No	20 (16.5)	33 (22.6)		10 (11.5)	39 (14.2)		13 (15.5)	88 (28.2)	
Tsunami									
Yes	11 (9.1)	19 (13.0)	0.312	37 (42.5)	68 (24.8)	<0.01	19 (22.6)	45 (14.4)	0.07
No	110 (90.9)	127 (87.0)		50 (57.5)	206 (75.2)		65 (77.4)	267 (85.6)	
Nuclear accident									
Yes	114 (94.2)	137 (93.8)	0.897	25 (28.7)	54 (19.7)	0.076	3 (3.6)	297 (95.2)	0.629
No	7 (5.8)	9 (6.2)		62 (71.3)	220 (80.3)		81 (96.4)	15 (4.8)	
Liquefaction									
Yes	2 (1.7)	3 (2.1)	0.809	3 (3.4)	6 (2.2)	0.512	26 (31.0)	63 (20.2)	<0.05
No	119 (98.3)	143 (97.9)		84 (96.6)	268 (97.8)		58 (69.0)	249 (79.8)	
Harmful rumors									
Yes	29 (24.0)	16 (11.0)	<0.01	28 (32.2)	32 (13.6)	<0.01	6 (7.1)	16 (5.1)	0.474
No	92 (76.0)	130 (89.0)		59 (67.8)	226 (82.5)		78 (92.9)	296 (94.9)	
Content of damage									
Own injuries/illness									
Yes	26 (21.5)	11 (7.5)	<0.01	11 (12.6)	5 (1.8)	<0.01	2 (2.5)	1 (0.3)	0.054
No	95 (78.5)	135 (92.5)		76 (87.4)	270 (98.2)		79 (97.5)	298 (99.7)	
Death of/missing family members or friends									
Yes	34 (28.1)	22 (15.1)	<0.01	7 (8.0)	11 (4.0)	0.13	4 (4.9)	1 (0.3)	<0.05
No	87 (71.9)	124 (84.9)		80 (92.0)	264 (96.0)		77 (95.1)	298 (99.7)	
Complete destruction of one’s house									
Yes	13 (10.7)	11 (7.5)	0.361	8 (9.2)	12 (3.7)	<0.01	1 (1.2)	4 (1.3)	0.942
No	108 (89.3)	135 (92.5)		79 (90.8)	269 (97.8)		80 (98.8)	295 (98.7)	
Partial destructions of one’s house									
Yes	45 (37.2)	59 (40.4)	0.591	34 (39.1)	102 (37.1)	0.738	26 (32.1)	53 (17.7)	<0.01
No	76 (62.8)	87 (59.6)		53 (60.9)	173 (62.9)		55 (67.9)	246 (82.3)	
Losing a job									
Yes	48 (39.7)	57 (39.0)	0.917	9 (10.3)	15 (5.5)	0.11	3 (3.7)	2 (0.7)	<0.05
No	73 (60.3)	89 (61.0)		78 (89.7)	260 (94.5)		78 (96.3)	297 (99.4)	
Family separation/discord									
Yes	60 (49.6)	49 (33.6)	<0.01	6 (6.9)	3 (1.1)	<0.01	4 (4.9)	6 (2.0)	0.144
No	61 (50.4)	97 (66.4)		81 (93.1)	272 (98.9)		77 (95.1)	293 (98.0)	
Causes of stress in the past month									
Economic problems									
Yes	30 (24.8)	22 (15.1)	<0.05	34 (40.0)	53 (20.2)	<0.01	28 (35.0)	44 (14.9)	<0.01
No	91 (75.2)	124 (84.9)		51 (60.0)	210 (79.8)		52 (65.0)	252 (85.1)	
Work-related problems									
Yes	29 (24.0)	18 (12.3)	<0.05	26 (30.6)	201 (76.4)	0.196	43 (53.8)	72 (24.3)	<0.01
No	92 (76.0)	128 (87.7)		59 (69.4)	62 (23.6)		37 (46.3)	224 (75.7)	
Neighborhood problems									
Yes	36 (29.8)	12 (8.2)	<0.01	9 (10.6)	9 (3.4)	<0.05	3 (3.8)	8 (2.7)	0.622
No	85 (70.2)	134 (91.8)		76 (89.4)	254 (96.6)		77 (96.3)	288 (97.3)	
Worries about one’s hometown/disaster									
Yes	88 (72.7)	51 (34.9)	<0.01	34 (40.0)	31 (11.8)	<0.01	12 (15.0)	11 (3.7)	<0.01
No	33 (27.3)	95 (65.1)		51 (60.0)	232 (88.2)		68 (85.0)	285 (96.3)	

**Table 4:** Logistic regression analysis of characteristics of the participants and depression as severe psychological distress.

	Fukushima Prefecture				Kitaibaraki City				Kamisu City			
	*p*-Value	OR	95% CI		*p*-Value	OR	95% CI		*p*-Value	OR	95% CI	
Age ≥ 60 (ref. 20–59)	0.142	1.85	0.81	4.21	0.085	0.46	0.19	1.12	0.056	0.27	0.07	1.03
Gender Female (ref. Male)	0.050	2.04	1.00	4.15	0.178	0.56	0.24	1.30	0.353	0.65	0.27	1.61
Causes of damage												
Tsunami					0.528	1.33	0.55	3.19				
Content of damage												
Own injuries/illness	0.000	5.73	2.53	12.99	0.079	3.55	0.87	14.53				
Death of/missing of family members or friends					0.023	4.93	1.25	19.42				
Partial destructions of one’s house					0.052	2.30	0.99	5.32				
Losing a job					0.279	2.03	0.56	7.32				
Causes of stress in the past month												
Economic problems					0.100	2.04	0.87	4.77	0.004	3.82	1.53	9.57
Work-related problems	0.002	4.23	1.69	10.57					0.368	1.53	0.61	3.87
Neighborhood problems	0.343	1.50	0.65	3.44	0.131	2.85	0.73	11.07				
Worries about one’s hometown/disaster	0.000	4.36	1.95	9.73	0.001	4.43	1.80	10.90	0.016	4.80	1.34	17.21
OR, Odds Ratio; CI, Confidence Interval												

**Table 5:** Logistic regression analysis of characteristics of the participants and post-traumatic stress symptoms.

	Fukushima Prefecture				Kitaibaraki City				Kamisu City			
	*p*-Value	OR	95% CI		*p*-Value	OR	95% CI		*p*-Value	OR	95% CI	
Age ≥ 60 (ref. 20–59)	0.894	0.95	0.47	1.92	0.814	1.07	0.61	1.90	0.932	0.97	0.49	1.92
Gender Female (ref. Male)	0.781	0.92	0.50	1.69	0.342	1.32	0.75	2.31	0.548	1.19	0.68	2.06
Causes of damage												
Earthquake									0.194	1.62	0.78	3.35
Tsunami					0.239	1.43	0.79	2.58				
Liquefaction									0.390	1.32	0.70	2.46
Harmful rumors	0.014	2.60	1.22	5.55	0.180	1.54	0.79	2.58				
Content of damage												
Own injuries/illness	0.004	3.61	1.52	8.57	0.064	3.33	0.93	11.88				
Death of/missing of family members or friends	0.270	1.49	0.73	3.05								
Complete destruction of one’s house					0.020	4.77	1.28	17.69				
Partial destructions of one’s house									0.155	1.60	0.84	3.06
Family separation/discord	0.051	1.84	1.00	3.40	0.079	4.04	0.85	19.26				
Causes of stress in the past month												
Economic problems	0.462	1.32	0.63	2.73	0.105	1.68	0.90	3.12	0.004	2.46	1.33	4.56
Work-related problems	0.339	1.52	0.65	3.57					0.001	2.69	1.48	4.91
Neighborhood problems	0.001	3.69	1.67	8.17	0.544	1.44	0.44	4.73				
Worries about one’s hometown/disaster	0.000	4.13	2.29	7.47	0.000	3.49	1.81	6.72	0.003	4.26	1.61	11.24
OR, Odds Ratio; CI, Confidence Interval												

## Data Availability

Data for this study is available on reasonable request from the corresponding author.

## References

[1] Measures Taken by the National Police Agency and Damage Conditions of the 2011 Tōhoku Earthquake and Tsunami. https://www.npa.go.jp/news/other/earthquake2011/pdf/higaijokyo.pdf.

[2] Deaths Due to the Great East Japan Earthquake from Vital Statistics. https://www.mhlw.go.jp/toukei/saikin/hw/jinkou/kakutei11/dl/14_x34.pdf.

[3] Change in the Number of Evacuees. http://www.reconstruction.go.jp/topics/main-cat2/sub-cat2-1/20181228_hinansha_suii.pdf.

[4] Steps for Revitalization in Fukushima Prefecture. http://www.pref.fukushima.lg.jp/uploaded/attachment/518132.pdf.

[5] The Number of Evacuees in Japan. https://www.reconstruction.go.jp/topics/main-cat2/sub-cat2-1/20161028_hinansha.pdf.

[6] Armenian H.K., Morikawa M., Melkonian A.K., Hovanesian A.P., Haroutunian N., Saigh P.A., Akiskal K., Akiskal H.S. (2000). Loss as a determinant of PTSD in a cohort of adult survivors of the 1988 earthquake in Armenia: Implications for policy. Acta Psychiatr. Scand..

[7] Maya-Mondragón J., Sánchez-Román F.R., Palma-Zarco A., Aguilar-Soto M., Borja-Aburto V.H. (2019). Prevalence of Post-traumatic Stress Disorder and Depression After the 19 September 2017 Earthquake in Mexico. Arch. Med. Res..

[8] Kılıç C., Ulusoy M. (2003). Psychological effects of the November 1999 earthquake in Turkey: An epidemiological study. Acta Psychiatr. Scand..

[9] Chan C.L.W., Wang C.-W., Qu Z., Lu B.Q., Ran M.-S., Ho A.H.Y., Yuan Y., Zhang B.Q., Wang X., Zhang X. (2011). Posttraumatic stress disorder symptoms among adult survivors of the 2008 Sichuan earthquake in China. J. Trauma. Stress.

[10] Record Magazine of the Great East Japan Earthquake. https://www.pref.ibaraki.jp/seikatsukankyo/bousaikiki/bousai/kirokushi/kirokushihp.html.

[11] Grimm A., Hulse L., Preiss M., Schmidt S. (2012). Post- and peritraumatic stress in disaster survivors: An explorative study about the influence of individual and event characteristics across different types of disasters. Eur. J. Psychotraumatol..

[12] Reifels L., Pietrantoni L., Prati G., Kim Y., Kilpatrick D.G., Dyb G., Halpern J., Olff M., Brewin C.R., O’Donnell M. (2013). Lessons learned about psychosocial responses to disaster and mass trauma: An international perspective. Eur. J. Psychotraumatol..

[13] Sodeyama N., Tachikawa H., Takahashi S., Aiba M., Haraguchi Y., Arai T. (2022). The Mental Health of Long-Term Evacuees outside Fukushima Prefecture after the Great East Japan Earthquake. Tohoku J. Exp. Med..

[14] M 9.1-2011 Great Tohoku Earthquake, Japan. https://earthquake.usgs.gov/earthquakes/eventpage/official20110311054624120_30/region-info.

[15] Kessler R.C., Barker P.R., Colpe L.J., Epstein J.F., Gfroerer J.C., Hiripi E., Howes M.J., Normand S.-L.T., Manderscheid R.W., Walters E.E. (2003). Screening for serious mental illness in the general population. Arch. Gen. Psychiatry.

[16] Furukawa T.A., Kawakami N., Saitoh M., Ono Y., Nakane Y., Nakamura Y., Tachimori H., Iwata N., Uda H., Nakane H. (2008). The performance of the Japanese version of the K6 and K10 in the World Mental Health Survey Japan. Int. J. Methods Psychiatr. Res..

[17] Weiss D.S., Wilson J.P., Keane T.M. (2004). The Impact of Event Scale-Revised.

[18] Horowitz M., Wilner N., Alvarez W. (1979). Impact of Event Scale: A measure of subjective stress. Psychosom. Med..

[19] Asukai N., Kato H., Kawamura N., Kim Y., Yamamoto K., Kishimoto J., Miyake Y., Nishizono-Maher A. (2002). Reliability and validity of the Japanese-language version of the impact of event scale-revised (IES-R-J): Four studies of different traumatic events. J. Nerv. Ment. Dis..

[20] Creamer M., Bell R., Failla S. (2003). Psychometric properties of the Impact of Event Scale—Revised. Behav. Res. Ther..

[21] Kessler R.C., Galea S., Gruber M.J., Sampson N.A., Ursano R.J., Wessely S. (2008). Trends in mental illness and suicidality after Hurricane Katrina. Mol. Psychiatry.

[22] Sato S., Ishida I., Hattori K., Ota M. (2016). The psychiatric characteristics of evacuees from Fukushima prefecture in Kitaibaraki city after the Great East Japan Earthquake. Jpn. J. Clin. Psychiatry.

[23] Slovic P. (1987). Perception of risk. Science.

[24] Neria Y., Nandi A., Galea S. (2008). Post-traumatic stress disorder following disasters: A systematic review. Psychol. Med..

[25] Tang B., Liu X., Liu Y., Xue C., Zhang L. (2014). A meta-analysis of risk factors for depression in adults and children after natural disasters. BMC Public Health.

[26] Katz C.L., Pellegrino L., Pandya A., Ng A., DeLisi L.E. (2002). Research on psychiatric outcomes and interventions subsequent to disasters: A review of the literature. Psychiatry Res..

